# Use of Antimicrobials among Suspected COVID-19 Patients at Selected Hospitals, Bangladesh: Findings from the First Wave of COVID-19 Pandemic

**DOI:** 10.3390/antibiotics10060738

**Published:** 2021-06-18

**Authors:** Syeda Mah-E-Muneer, Md. Zakiul Hassan, Md. Abdullah Al Jubayer Biswas, Fahmida Rahman, Zubair Akhtar, Pritimoy Das, Md. Ariful Islam, Fahmida Chowdhury

**Affiliations:** 1 Infectious Diseases Division, International Centre for Diarrhoeal Disease Research, Bangladesh (icddr,b), Dhaka 1212, Bangladesh; zhassan@icddrb.org (M.Z.H.); jubayer.biswas@icddrb.org (M.A.A.J.B.); fahmida.rahman@icddrb.org (F.R.); zakhtar@icddrb.org (Z.A.); pritimoy@icddrb.org (P.D.); arif@icddrb.org (M.A.I.); fahmida_chow@icddrb.org (F.C.); 2 Nuffield Department of Medicine, University of Oxford, Oxford OX1 2JD, UK

**Keywords:** COVID-19, suspected COVID-19, SARS-CoV-2, antimicrobial use, antibiotic, Bangladesh

## Abstract

Antimicrobials are empirically used in COVID-19 patients resulting in increased antimicrobial resistance. Our objective was to assess antimicrobial use among suspected COVID-19 in-patients. From March to August 2020, we collected data from in-patients of 12 tertiary-level hospitals across Bangladesh. We identified suspected COVID-19 patients; collected information on antimicrobial received within 24 h before and on hospitalization; tested nasopharyngeal swab for SARS-CoV-2 using rRT-PCR. We used descriptive statistics and a regression model for data analysis. Among 1188 suspected COVID-19 patients, 69% were male, 40% had comorbidities, and 53% required oxygen. Antibiotics were used in 92% of patients, 47% within 24 h before, and 89% on admission. Patients also received antiviral (1%) and antiparasitic drugs (3%). Third-generation cephalosporin use was the highest (708; 60%), followed by macrolide (481; 40%), and the majority (853; 78%) who took antibiotics were SARS-CoV-2 negative. On admission, 77% mild and 94% moderately ill patients received antibiotics. Antibiotic use on admission was higher among severely ill patients (AOR = 11.7; 95% CI: 4.5–30.1) and those who received antibiotics within 24 h before hospital admission (AOR = 1.6; 95% CI: 1.0–2.5). Antimicrobial use was highly prevalent among suspected COVID-19 in-patients in Bangladesh. Initiating treatment with third-generation cephalosporin among mild to moderately ill patients was common. Promoting antimicrobial stewardship with monitoring is essential to prevent blanket antibiotic use, thereby mitigating antimicrobial resistance.

## 1. Introduction

Different antimicrobials are frequently being administered to treat the novel coronavirus disease (COVID-19) even though it is a viral illness [1,2]. Due to the ambiguity of an effective treatment strategy, an empirical approach by the healthcare personnel (HCP) is to prescribe varieties of antibiotics for treating COVID-19 patients irrespective of the laboratory parameters [3]. The decision behind starting antibiotics is mostly based on clinical presentations that are similar to bacterial pneumonia [4,5]. Concern for community-acquired pneumonia, nosocomial bacterial superinfection in longer hospitalized patients, and high mortality due to COVID-19 among risk groups such as age over 60 years and underlying medical conditions has made the HCP consider antibiotics as a treatment strategy [3,6,7,8]. Uses of antibiotics for treating COVID-19 patients were found up to 72% around the globe [5,6,7,8,9], whereas bacterial co-infection was only 1–16% among those patients who received antibiotics [9,10,11,12,13]. Broad-spectrum antibiotics were commonly used in order to cover a wide range of bacteria [4,6,7,8,9]. Moreover, antivirals [8,14,15,16], antimalarial, such as hydroxychloroquine [17], and antiparasitic drugs, e.g., ivermectin [18,19], were also used for treating COVID-19. COVID-19 treatment has led long term and expanded use of antibiotics. Empiric antibiotic treatment is silently and gradually heading to higher antimicrobial resistance (AMR) and becoming a global threat [2,20,21,22,23]. As a result, an increased number of morbidity and mortality is likely to occur during and beyond the pandemic. World Health Organization (WHO) expressed concern that the AMR situation, which is already an urgent challenge of this time, will get worse due to the COVID-19 pandemic [24].

Bangladesh, a densely populated low-middle income country in Southeast Asia, reported 529,687 confirmed COVID-19 cases with 7950 deaths as of 20 January 2021, from the first detection on 8 March 2020 [25]. Most of the public hospitals lack microbiology laboratory and healthcare workforce but remain overcrowded with patients and their attendants [26,27]. During the early COVID-19 pandemic situation, very few facilities were equipped to test for SARS-CoV-2. It caused a long queue for testing and huge pressure on laboratory facilities, resulting in delayed test reports up to 5–10 days [28]. It became a barrier for testing and healthcare-seeking for people having symptoms [28,29]. To get rid of this illness, people with suspected COVID-19 took self-medication without performing any test [30,31]. These existing situations raised the concern for the overuse of antibiotics in the COVID-19 pandemic. Guidance on clinical management and treatment of COVID-19 cases by WHO is available [32]. Adopting this guidance and modifying considering the local context, Bangladesh introduced national guidelines on clinical management of COVID-19 [33]. To promote optimal use and reduce overprescription of antibiotics, WHO also recommends AWaRE (Access, Watch, Reserve)—a new tool on antibiotic classification [34]. However, it was uncertain that how closely these guidelines were followed. Moreover, there is a scarcity of data regarding antimicrobial use among hospital admitted suspected COVID-19 patients in Bangladesh. Available studies reported antibiotic use among confirmed COVID-19 patients, suggesting a wide range of antibiotic use during the COVID-19 pandemic [35,36]. Considering the scarcity of antimicrobial data in this overstressing COVID-19 pandemic situation in Bangladesh, we assessed the proportion of antimicrobial use and factors associated with antibiotic use among suspected COVID-19 patients.

## 2. Results

We included 1188 suspected COVID-19 patients in the analysis. Among them, 17% (*n* = 205) were from COVID-19 sentinel surveillance and 83% (*n* = 983) from hospital-based influenza surveillance.

### 2.1. Demographic and Clinical Characteristics

Among 1188 suspected COVID-19 patients, the median age was 34 years (IQR: 2–56), and the majority were between 19 and 59 years (41%), male (69%), and reported being non-smokers (79%) (Table 1). Only 1% were healthcare workers, and 0.7% were pregnant. 

Table 2 shows the clinical characteristics of the enrolled patients. One or more comorbidities were found in 40% of patients, with 208 (17%) having diabetes, 182 (15%) having hypertension, and 169 (14%) having chronic lung diseases, including asthma, emphysema and chronic obstructive pulmonary disease (COPD). Overall, 628 (53%) patients required oxygen, and 7 (1%) required ICU or ventilation support after admission.

### 2.2. Proportion of Antibiotic Use (before and on Hospital Admission)

The overall proportion of antibiotic use was 92% among suspected COVID-19 patients. Among the suspected COVID-19 patients, 33 (3%) reported taking antibiotics only within 24 h before admission, 528 (44%) received antibiotics only within 24 h after admission, and 529 (45%) received antibiotics at both points—before and after admission. The proportion was higher for patients aged 19–59 years, patients with cough, and moderately ill patients (pneumonia) (Table 1 and Table 2). 

### 2.3. Healthcare Sought within Last Two Weeks Prior to Hospital Admission for Current Illness

Among the 205 patients from COVID-19 surveillance, the overall antibiotic use was 73% (*n* = 150); 40 (20%) patients reported taking antibiotics within 24 h before hospital admission. Of these 40 patients, 15 (37%) had a history of admission in a healthcare facility, 15 (37%) visited registered healthcare providers, 8 (20%) visited informal healthcare providers, such as non-medically trained traditional healthcare provider or local drug-sellers, and the remaining 2 (5%) had no history of seeking any healthcare but received a self-prescribed antibiotic. 

### 2.4. Different Class of Antibiotic Used among SARS-CoV-2 Positive and Negative Patients before and on Hospital Admission

Among all suspected COVID-19 patients, the most frequently reported antibiotic class was cephalosporin (64%), particularly third-generation cephalosporin (60%), a Watch group antibiotic (Table 3). The second most frequently reported antibiotic class was macrolide (40%), where azithromycin (Watch group) was mostly (98%) used. 

Before admission, the most frequently used antibiotic class was macrolide (327; 27%), followed by cephalosporin (187; 16%). However, on admission, the antibiotic choice was the opposite; cephalosporin (727; 61%) followed by macrolide (265; 22%) (Table 3). Before admission, 32 (3%) suspected patients reported using two antibiotics, and on admission, 322 (27%) received two to four classes of antibiotics at the same time. Of those who received antibiotics either before or on admission, the majority (78%) were SARS-CoV-2 negative.

According to WHO AWaRe classification, the Watch group antibiotics were mostly used before (43%) as well as on admission (80%) (Table 3).

### 2.5. Antibiotic Use among Patients with Different COVID-19 Severity on Admission

Antimicrobial use among patients with four types of COVID-19 disease severity—mild–critical—on admission is shown in Table 4. The third-generation cephalosporin was mostly used among all four types of patients. In contrast, the first-generation cephalosporin was not used in any patient. The second most used antibiotic was macrolide among mild, moderate and severe patients and penicillin in critical patients. Among mild and moderately ill patients, 4% and 2%, respectively, received carbapenem, particularly meropenem (Watch group). All four categories of patients, with the majority from mild, reported receiving Reserve group antibiotics, particularly oxazolidinones (i.e., linezolid), on admission. 

Antibiotics used among suspected COVID-19 patients in different wards and ICU on admission are shown in Table A1.

### 2.6. Antiviral Use

None of the suspected COVID-19 patients took antiviral before admission; rather, it was used among 16 (1.4%) patients on admission (Table 3). Among them, 14 (87%) were SARS-CoV-2 test negative. Half of the patients who received antiviral had a mild illness, whereas only one from the critical category received antiviral (Table 4). The most frequently used antiviral was favipiravir (1%). 

### 2.7. Antiparasitic Drug Use

Overall ivermectin use was reported from 3% of the suspected COVID-19 patients; three patients used it before admission, and 30 received it on admission (Table 3). Ivermectin use was higher in severe COVID-19 patients on admission (Table 4).

### 2.8. Steroid Use (Other Medication)

A non-antimicrobial drug, steroid use was also reported before and on admission. Among the suspected COVID-19, a steroid was used in 105 (9%) patients; of those, 88 (84%) were SARS-CoV-2 test negative. Of those who received steroids, more than half (54%) had a mild illness, and about one-third (36%) had asthma, COPD, or sepsis. Before admission, only hydrocortisone was reported (*n* = 13), and on admission, hydrocortisone (*n* = 56) and dexamethasone (*n* = 37) were the most frequently used steroids among suspected COVID-19 patients. 

### 2.9. Potential Factors Associated with Antibiotic Use on Hospital Admission

The factors associated with antibiotic use among suspected COVID-19 patients after admission are shown in Table 5. In multivariable analysis, disease severity and antibiotic use within 24 h before hospital admission were found to be significantly associated with antibiotic use among study patients. Patients who had a severe and moderate level of illness were 11.7 and 3.6 times more likely (95% CI: 4.5–30.1, 2.2–6.0) to use antibiotics than the mild category, respectively. Similarly, a higher probability of antibiotic use was found among those who received antibiotics within 24 h before hospital admission (AOR = 1.6; 95% CI: 1.0–2.5).

## 3. Discussion

This study assessed the proportion of antimicrobial use among patients with COVID-19 symptoms. Our study findings suggest that antibiotic use was as high as 92% in treating patients before the COVID-19 test report was available. A study conducted in the early pandemic period at Wuhan, China reported, all suspected COVID-19 hospitalized cases were treated with empirical antibiotics [14]. A similar treatment strategy was found in a recent Bangladeshi study where all confirmed COVID-19 patients received one broad-spectrum antibiotic on admission in a tertiary care hospital [35]. Our study also found a higher number of antibiotic uses among suspected COVID-19 patients. Of whom, three-fourths were SARS-CoV-2 negative. Clinicians empirically initiated antibiotics based on the patient’s clinical presentation on admission as the COVID-19 test report was not available due to the long turnaround time to receive the COVID-19 test result. 

Our study findings show that most of the mild and moderately ill suspected COVID-19 patients received antibiotics on admission. This proportion was higher compared to a study conducted among adult hospitalized patients, where 24% mild and 60% moderate patients with COVID-19 received antibiotics on admission [6]. Prescribed antibiotic use found in our study was not adherent with the national [33] as well as the WHO guidelines on COVID-19 clinical management. In these guidelines, antibiotic use as prophylaxis for mild patients is strongly discouraged. However, for moderate patients, antibiotics are recommended for clinically suspected bacterial infection. Our study patients did not have any bacterial culture report at the time of initiating antibiotics on admission. This indicates non-compliance by the clinicians while prescribing antibiotics on admission. Complete adherence to guidelines among clinicians is highly required for optimal use of antibiotics. 

WHO-AWaRe classification, developed in 2019 for selection and use of antibiotics, recommends using only Access antibiotics but not Watch or Reserve antibiotics [34,37]. In our study, we found Watch antibiotics were highly used. We found no use of first-generation cephalosporin (Access antibiotics) in any patient; rather, broad-spectrum antibiotics, particularly third-generation cephalosporin (Watch antibiotics), a critically important antimicrobial, was the major choice for empirical approach. This finding is consistent with previous studies where third-generation cephalosporin use was found higher for COVID-19 patients [1,6,10]. Other broad-spectrum antibiotics such as piperacillin/tazobactam, vancomycin, carbapenems, tigecycline, and combinations with fluoroquinolones were also used for treating COVID-19 patients [4,6,7,8,9], which are similarly found in our study. Reserve antibiotics such as linezolid were also found to be used even in mild and pediatric patients in our study. However, according to our study findings antibiotic use did not follow the WHO guideline, and it suggests an urgent requirement for strategies, such as frequent refresher training on the AWaRe tool. This might help clinicians prescribe safe and effective rational antibiotics with proper judgment. This widespread use of antibiotics during this COVID-19 pandemic is an alarming threat to antimicrobial resistance. Introducing antibiotic stewardship programs (ASP) with the formation of an active ASP committee in hospitals could be considered to reduce the irrational use of antibiotics and to improve rational antibiotic prescribing practices.

Antiviral use was found much lower compared to antibiotic use among suspected COVID-19 patients in our study. Previous studies reported similar lower use of antivirals such as oseltamivir, ganciclovir, remdesivir, lopinavir, favipiravir and ritonavir [8,14,15,16]. We found favipiravir was mostly used among all antivirals on admission. This finding goes against WHO-recommendation where it was mentioned not to administer favipiravir as treatment or prophylaxis for COVID-19, outside the context of clinical trials [32]. However, our national guideline on clinical management of COVID-19 recommends favipiravir for moderately-ill COVID-19 patients [33]. 

Ivermectin is a potential drug against COVID-19 infection yet to be approved and recommended only for clinical trial [18,19,38]. In our study, we observed using this anti-parasitic drug for COVID-19 treatment. One possible reason for using could be the antiviral property of this drug [38]. However, clinicians should hold back its use until it is fully recommended for the treatment of COVID-19 patients. 

The application of steroids in COVID-19 is debated as its role is still unproven [39,40]. WHO recommends not to use systemic corticosteroids routinely for the treatment of viral pneumonia unless indicated, such as acute exacerbation of asthma, COPD, septic shock, or ARDS [32]. Despite the restriction, 9% of patients in our study reported receiving systemic corticosteroids; and two-third of them did not have asthma, COPD, septic shock or ARDS. The reason for using steroids could be that in our national guideline, oral methylprednisolone has been recommended in moderate COVID-19 cases in particular circumstances [33]. However, we observed that mild patients also received systemic corticosteroids on admission, which is not in accordance with the local guideline. Clinicians should strictly follow the guideline while prescribing COVID-19 patients, considering the threat that steroid use can impose [40]. 

We also found patients who received antibiotics within 24 h before hospital admission are two times more likely to receive antibiotics on hospital admission. It was likely because the physician wanted to complete the course or changed the antibiotic on admission.

In a subset of patients, we found 25% of patients who took antibiotics before admission reported visiting informal (unregistered) healthcare providers and having self-prescribed antibiotics—a common practice in Bangladesh [41]. People in Bangladesh, regardless of socio-economic status and education, took over-the-counter medicines and even antibiotics without consulting any qualified healthcare providers [41,42]. This practice of self-medication increased up to 88% during the COVID-19 pandemic [30,31]. Taking medicine without performing a COVID-19 test for symptoms, such as fever, sore throat, or cough, was found to be common; ivermectin (77%) and azithromycin (54%) were the most frequently used self-medicated drugs in Bangladesh during the COVID-19 pandemic [30]. Anxiety due to pandemic, avoiding going out during lockdown, high self-awareness, inability to access telemedicine, a suggestion from family members, friends, and local drug sellers, a low-cost alternate of treatment, low ratio between doctor and patient, and easy availability of drugs may have led to self-medication [30,43]. Educational campaigns immediately need to be strengthened to make the community aware of the consequences of self-medicated antibiotics. Adopting prescription-only status by all the pharmacies with strict monitoring by the government may reduce self-medication for antibiotics.

Our study had certain limitations. We only documented antimicrobial use among suspected COVID-19 patients within the first 24 h of hospital admission but not after 24 h of admission. Clinicians might have introduced, discontinued or switched to alternative antibiotics on a later date even after receiving the COVID-19 test results. Moreover, we did not collect information on ultimate diagnosis, including bacterial co-infection among patients. Therefore, we missed assessing the appropriateness of empirical antibiotic treatment. We also did not collect healthcare-seeking information before hospital admission from hospital-based influenza surveillance due to logistical challenges in a routine surveillance system.

However, this study findings documented an early assessment of the antimicrobial use among suspected COVID-19 patients 24 h before and 24 h after hospital admission. This study provides a first-hand picture with comprehensive information on antimicrobial treatment in patients at the suspected COVID-19 phase before receiving the COVID-19 test result. Our surveillance hospitals are situated in 12 different districts at diverse geographic locations across Bangladesh.

## 4. Materials and Methods

### 4.1. Study Site and Study Population

**Figure 1:**
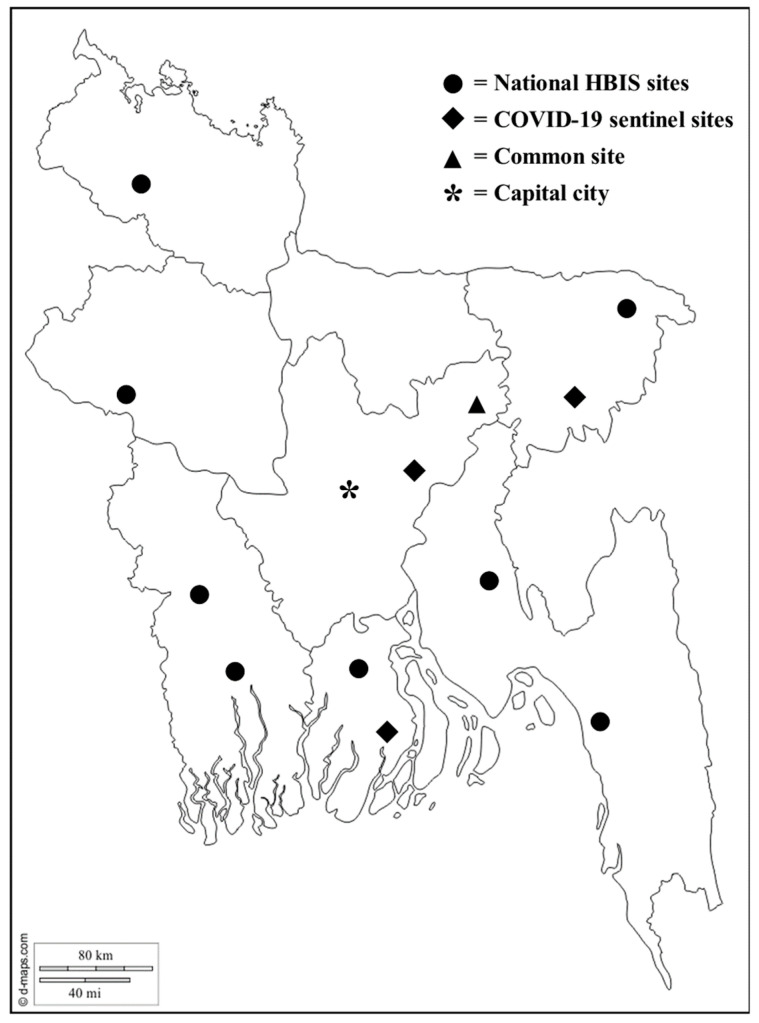
Location of the 12 surveillance hospitals across Bangladesh.

We collected data from two hospital-based surveillance platforms at 12 selected hospitals at different geographical locations across Bangladesh from 1 March to 31 August 2020 (Figure 1). Our study population was patients of all age groups admitted to the study wards. Both the surveillance systems were conducted in collaboration with the Institute of Epidemiology, Disease Control and Research (IEDCR) of the Government of Bangladesh (GoB).

### 4.2. Surveillance Activities

#### 4.2.1. COVID-19 Sentinel Surveillance

To identify COVID-19 among patients attending hospitals with an acute respiratory infection, we conducted the surveillance at medicine and pediatric wards, COVID-19 isolation unit, and intensive care unit (ICU) in 4 hospitals—3 district level public and 1 tertiary level private hospital. We enrolled patients from 10 June to 31 August 2020, from this platform.

#### 4.2.2. Hospital-Based Influenza Surveillance (HBIS)

To identify the respiratory viruses among hospitalized patients with severe acute respiratory infections defined as a patient with history of fever and cough within the last 10 days (adopted from WHO), this nationally representative surveillance was initiated from May 2007 and still ongoing at 9 tertiary level (7 public and 2 private) hospitals [44,45]. We enrolled patients admitted to medicine and pediatric wards, COVID-19 isolation unit, and coronary care unit (CCU) from 1 March to 31 August 2020. One hospital was common in both the surveillance platforms, and we considered patients from that hospital once.

### 4.3. Patient Enrolment and Data Collection

We followed the Bangladesh government-defined case definition of suspected COVID-19: any patient having one or more of the symptoms, including fever, cough, sore throat, or difficulty breathing within the last 7 days [33]. We enrolled hospitalized patients who met the inclusion criteria and provided informed written consent by the patient or caregiver. For outcome, we collected a list of antimicrobials, e.g., antibiotic, antiviral, antimalarial, antifungal, antiparasitic drugs, and other medication prescribed on admission from the patient files within 24 h of admission. Trained field staff interviewed the patient or guardian on antimicrobial use within 24 h before hospital admission for current illness episode. They also collected data on relevant factors that may be associated with antibiotic use, such as demographics and clinical information, including vital signs, complete blood count report, and chest X-ray findings (if available); comorbid conditions and provisional diagnosis on admission. From patients of the COVID-19 surveillance platform, the staff additionally collected information on whether any type of healthcare was sought in two weeks preceding current hospital admission.

### 4.4. Sample Collection and Laboratory Testing

Followed by data collection, we collected a nasopharyngeal swab. The samples were kept in viral transport media (VTM) and shipped to virology laboratory at icddr,b in Dhaka city, maintaining cool chain for testing SARS-CoV-2 using a one-step real-time reverse transcription polymerase chain reaction (rRT-PCR).

### 4.5. Disease Severity Category among Suspected COVID-19 Patients

COVID-19 disease severity was categorized as mild, moderate, severe, and critical using WHO’s COVID-19 clinical management guideline (Table A2).

### 4.6. Data Analysis

**Figure 2:**
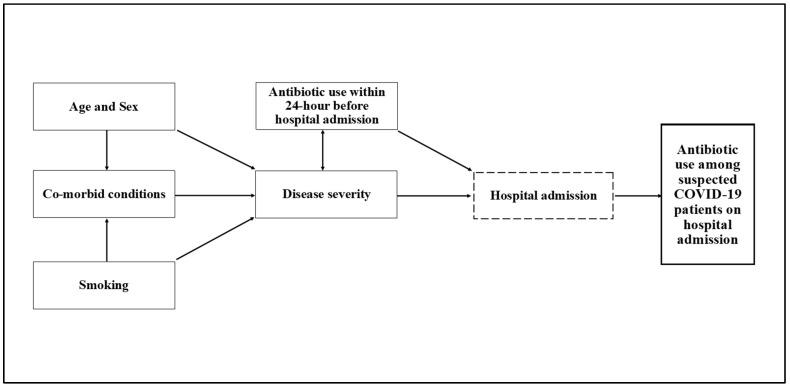
Conceptual framework for the causal relationship between antibiotic use and explanatory variables, including patient age, sex, comorbid conditions, smoking status, disease severity, and antibiotic use within 24 h before hospital admission.

We conducted data analysis using Stata 15 software (Stata Corp. 2017. Stata Statistical Software: Release 13. College Station, TX, USA: Stata Corp LP.). We used frequency and percentage to summarize the background characteristics of the enrolled patients. We illustrated the use of antimicrobials among suspected COVID-19 patients by reporting frequency and percentage. We also performed bivariable binary logistic regression to estimate the crude relationship between exploratory variables and the outcome of interest. We reported output as the unadjusted odds ratio (OR) with 95% CI and *p*-value. We utilized the conceptual framework technique, which displayed the potential causal pathway and link between antibiotic use after admission and explanatory variables, including patient age, sex, comorbid conditions, smoking status, disease severity, and antibiotic use before hospital admission to delineate the causal relationship (Figure 2) [46]. Technical details of the conceptual framework technique have been explained in Jewell et al. [47]. Variables with missing values, such as healthcare-seeking behavior before admission, were excluded from the model. Multivariable logistic regression was utilized to calculate the association of each explanatory variable with the outcome of interest by adjusting confounders and reported as adjusted odds ratio (AOR) with 95% CI and *p*-value, where *p* < 0.05 was considered as the statistical significance level. All tests were two-tailed and were considered significant at an alpha value of 5%. 

## 5. Conclusions

Antimicrobials, predominantly antibiotic use, was highly prevalent among hospital admitted suspected COVID-19 patients in Bangladesh. Initiating treatment with third-generation cephalosporin (Watch antibiotic) is common even in mild to moderate COVID-19 patients. These findings reflect that irrational use of antibiotics is being exercised without following the guideline. This blanket use of antibiotics in viral pandemic raises the concern for increasing long-term threat of AMR during and post COVID-19 pandemic period. It is of extreme importance to promote, standardize, and strengthen antimicrobial stewardship considering the key elements, including the adoption of WHO’s AWaRe tool with constant monitoring of adherence to the COVID-19 treatment guideline. Formative studies need to be conducted to better understand the drivers of prescribing antibiotics among clinicians.

## Figures and Tables

**Table 1:** The use of antibiotics by demographic characteristics among the suspected COVID-19 patients admitted at 12 selected hospitals in Bangladesh, March–August 2020.

Characteristics	Suspected COVID-19 Patients (*n* = 1188) *n* (%)	Antibiotic Use *n* (%)		
		Overall (*n* = 1090)	COVID-19 Sentinel Surveillance (*n* = 150)	Hospital-Based Influenza Surveillance (*n* = 940)
**Age in year**				
≤5	322 (27.1)	312 (28.6)	1 (0.7)	311 (33.1)
6–18	96 (8.1)	89 (8.2)	5 (3.3)	84 (8.9)
19–59	486 (40.9)	436 (40.0)	51 (34.0)	385 (40.9)
≥60	284 (23.9)	253 (23.2)	93 (62.0)	160 (17.0)
**Gender**				
Male	816 (68.7)	755 (69.3)	102 (68.0)	653 (69.5)
Female	372 (31.3)	335 (30.7)	48 (32.0)	287 (30.5)
**Type of ward**				
Medicine	676 (56.9)	633 (58.1)	94 (62.7)	539 (57.3)
Pediatrics	389 (32.7)	375 (34.4)	5 (3.3)	370 (39.4)
ICU	2 (0.2)	2 (0.2)	1 (0.7)	1 (0.1)
CCU	54 (4.5)	43 (3.9)	13 (8.7)	30 (3.2)
COVID-19 Isolation	67 (5.6)	37 (3.4)	37 (24.7)	0
**COVID-19 test result**				
Positive	257 (21.6)	237 (21.7)	44 (29.3)	193 (20.5)
Negative	931 (78.4)	853 (78.3)	106 (70.7)	747 (79.5)
**Health care worker**				
Yes	14 (1.2)	12 (1.1)	0	12 (1.3)
No	1174 (98.8)	1078 (98.9)	150 (100.0)	928 (98.7)
**Smoking**				
Smoker	250 (21.0)	220 (20.2)	53 (35.3)	167 (17.8)
Non-smoker	938 (79.0)	870 (79.8)	97 (64.7)	773 (82.2)

**Table 2:** The use of antibiotics by clinical characteristics among the suspected COVID-19 patients admitted at 12 selected hospitals in Bangladesh, March–August 2020.

Characteristics	Suspected COVID-19 Patients (*n* = 1188) *n* (%)	Antibiotic Use *n* (%)		
		Overall (*n* = 1090)	COVID-19 Sentinel Surveillance (*n* = 150)	Hospital-Based Influenza Surveillance (*n* = 940)
**Signs and symptoms on admission**				
Cough (dry/productive)	1097 (92.3)	1027 (94.2)	87 (58.0)	940 (100.0)
Fever	1063 (89.5)	996 (91.4)	56 (37.3)	940 (100.0)
Shortness of breath	845 (71.1)	797 (73.1)	67 (44.7)	730 (77.7)
Runny nose	405 (34.1)	380 (34.9)	9 (6.0)	371 (39.5)
Headache	382 (32.1)	348 (31.9)	29 (19.3)	319 (33.9)
Sore throat	225 (18.9)	210 (19.3)	18 (12.0)	192 (20.4)
Loss of smell or taste (*n* = 205)	69 (33.7)	49 (4.5)	49 (32.7)	0
Fever, cough and difficulty breathing	765 (64.4)	739 (67.8)	9 (6.0)	730 (77.7)
**Comorbidities**				
None	708 (59.6)	658 (60.4)	48 (32.0)	610 (64.9)
One or more	480 (40.4)	432 (39.6)	102 (68.0)	330 (35.1)
**COVID-19 disease severity (WHO category)**				
Mild (symptoms only)	436 (36.7)	357 (32.8)	109 (72.7)	248 (26.4)
Moderate (pneumonia)	389 (32.7)	374 (34.3)	33 (22.0)	341 (36.3)
Severe (severe pneumonia)	326 (27.4)	323 (29.6)	6 (4.0)	317 (33.7)
Critical (ARDS or Sepsis or septic shock or ICU/ventilation)	37 (3.1)	36 (3.3)	2 (1.3)	34 (3.6)
**Care seeking before admission (*n* = 205)**				
Visited any healthcare provider or facility within two weeks of hospital admission	160 (78.0)	114 (76.0)	114 (76.0)	0
**Death occurred in hospital**	69 (5.8)	66 (6.1)	13 (8.7)	53 (5.6)

**Table 3:** Antimicrobials used among suspected COVID-19 patients and SARS-CoV-2 positive and negative patients 24 h before and on hospital admission at 12 selected hospitals in Bangladesh, March–August 2020.

Antimicrobials *	Suspected COVID-19 Patients (*n* = 1188) *n* (%)	Within 24 h before Admission			On Admission		
		SARS-CoV-2 Positive (*n* = 257) *n* (%)	SARS-CoV-2 Negative (*n* = 931) *n* (%)	*p*-Value	SARS-CoV-2 Positive (*n* = 257) *n* (%)	SARS-CoV-2 Negative (*n* = 931) *n* (%)	*p*-Value
**Antibiotic**							
Antibiotics used in total	1090 (91.7%)	562 (47.3%)			1057 (89.0%)		
Cephalosporin	761 (64.1)	25 (9.7)	161 (17.3)	0.003	127 (49.4)	600 (64.4)	<0.001
First-generation	4 (0.3)	1 (0.4)	3 (0.3)	0.870	0	0	
Second-generation	69 (5.8)	5 (2)	13 (1.4)	0.523	7 (2.7)	47 (5.1)	0.113
Third-generation	708 (59.6)	19 (7.4)	145 (15.6)	0.001	120 (46.7)	554 (59.5)	<0.001
Fourth-generation	1 (0.1)	0	0	0	0	1 (0.1)	0.599
Macrolide	481 (40.5)	104 (40.5)	223 (24.0)	<0.001	85 (33.1)	180 (19.3)	<0.001
Penicillin	200 (16.8)	6 (2.3)	27 (2.9)	0.625	38 (14.8)	140 (15.0)	0.920
Aminoglycoside	114 (9.6)	1 (0.4)	6 (0.6)	0.636	7 (2.7)	102 (11.0)	<0.001
Quinolones	63 (5.3)	3 (1.2)	8 (0.9)	0.648	11 (4.3)	41 (4.4)	0.932
Tetracycline	76 (6.4)	10 (3.9)	14 (1.5)	0.016	17 (6.6)	39 (4.2)	0.104
Carbapenems	45 (3.8)	0	3 (0.3)	0.362	11 (4.3)	33 (3.5)	0.580
Oxazolidinone	12 (1.0)	0	0		3 (1.2)	9 (1.0)	0.776
Glycopeptides	10 (0.8)	0	0		2 (0.8)	8 (0.9)	0.900
Nitroimidazoles	13 (1.1)	0	1 (0.1)	0.599	3 (1.2)	9 (1.0)	0.776
**WHO AWaRe classification antibiotics**							
Access	370 (31.1)	68 (5.7)			329 (27.7)		
Watch	1016 (85.5)	514 (43.3)			949 (79.9)		
Reserve	12 (1.0)	0			12 (1.0)		
**Antiviral drug**							
Antiviral used in total	16 (1.4)	0			16 (1.4)		
Acyclovir	3 (0.3)	0	0	-	0	3 (0.3)	0.362
Adefovir	1 (0.1)	0	0	-	0	1 (0.1)	0.599
Zidovudine	1 (0.1)	0	0	-	0	1 (0.1)	0.599
Valacyclovir	3 (0.3)	0	0	-	1 (0.4)	2 (0.2)	0.622
Favipiravir	8 (0.7)	0	0	-	1 (0.4)	7 (0.7)	0.529
**Antiparasitic drug**							
Antiparasitic drug used in total	33 (2.8)	3 (0.3)			30 (2.5)		
Ivermectin	33 (2.8)	1 (0.4)	2 (0.2)	0.622	9 (3.5)	21 (2.7)	0.260

* Multiple response.

**Table 4:** Antimicrobials used on admission among suspected COVID-19 patients according to disease severity at 12 selected hospitals in Bangladesh, March–August 2020.

Antimicrobials	Disease Severity				
	Mild (*n* = 436) *n* (%)	Moderate (*n* = 389) *n* (%)	Severe (*n* = 326) *n* (%)	Critical (*n* = 37) *n* (%)	Total (*n* = 1188) *n* (%)
**Antibiotic**	334 (76.6)	367 (94.3)	321 (98.5)	35 (94.6)	1057 (89.0)
Cephalosporin	197 (45.2)	259 (66.6)	250 (76.7)	21 (56.8)	727 (61.2)
First-generation	0	0	0	0	0
Second-generation	10 (2.3)	30 (7.7)	10 (3.1)	4 (10.8)	54 (4.6)
Third-generation	187 (42.9)	231 (59.4)	239 (73.3)	17 (46.0)	674 (56.7)
Fourth-generation	0 (0)	0 (0)	1 (0.3)	0 (0)	1 (0.1)
Macrolide	88 (20.2)	98 (25.2)	74 (22.7)	5 (13.5)	265 (22.3)
Penicilin	48 (11)	59 (15.2)	63 (19.3)	8 (21.6)	178 (15.0)
Aminoglycoside	15 (3.4)	37 (9.5)	51 (15.6)	6 (16.2)	109 (9.2)
Quinolones	27 (6.2)	11 (2.8)	13 (4)	1 (2.7)	52 (4.4)
Tetracycline	18 (4.1)	16 (4.1)	16 (4.9)	6 (16.2)	56 (4.7)
Carbapenems	17 (3.9)	6 (1.5)	18 (5.5)	3 (8.1)	44 (3.7)
Oxazolidinones	4 (0.9)	3 (0.8)	2 (0.6)	3 (8.1)	12 (1.0)
Glycopeptides	4 (0.9)	2 (0.5)	2 (0.6)	2 (5.4)	10 (0.8)
Nitroimidazoles	9 (2.1)	2 (0.5)	0 (0)	1 (2.7)	12 (1.0)
**WHO AWaRe classification antibiotics**					
Access	82 (18.8)	106 (27.2)	122 (37.4)	19 (51.3)	329 (27.7)
Watch	287 (65.8)	341 (87.7)	293 (89.9)	28 (75.7)	949 (79.9)
Reserve	4 (0.9)	3 (0.8)	2 (0.6)	3 (8.1)	12 (1.0)
**Antiviral drug**					
Acyclovir	2 (0.5)	0	0	1 (2.7)	3 (0.2)
Adefovir	0	1 (0.3)	0	0	1 (0.1)
Zidovudine	1 (0.2)	0	0	0	1 (0.1)
Valacyclovir	3 (0.7)	0	0	0	3 (0.2)
Favipiravir	2 (0.5)	0	6 (1.8)	0	8 (0.7)
**Antiparasitic drug**					
Ivermectin	3 (0.7)	6 (1.5)	16 (4.9)	5 (13.5)	30 (2.5)

**Table 5:** Independently associated factors with antibiotic use on hospital admission among suspected COVID-19 patients at 12 selected hospitals in Bangladesh, March–August 2020.

	UOR (95% CI)	*p*-Value	AOR (95% CI)	*p*-Value
Disease severity ^1^				
Mild	Reference		Reference	
Moderate	5.1 (3.1–8.3)	<0.001	3.6 (2.2–6.0)	<0.001
Severe	19.6 (7.9–48.8)	<0.001	11.7 (4.5–30.1)	<0.001
Critical	5.3 (1.3–22.6)	0.023	3.4 (0.8–14.9)	0.098
Antibiotic use before hospital admission ^2^				
No	Reference		Reference	
Yes	3.0 (2.0–4.5)	<0.001	1.6 (1.0–2.5)	0.044

^1^ adjusted for age, sex, comorbid condition, smoking, antibiotic use before hospital admission, surveillance type; ^2^ adjusted for disease severity, surveillance type.

## Data Availability

The data presented in these surveillances are available on request from the corresponding author. The data are not publicly available due to privacy restrictions and icddr,b policy.

## Appendix A

**Table A1:** Antimicrobials used for suspected COVID-19 patients on admission in different wards, CCUs, and ICUs of 12 selected hospitals in Bangladesh, March–August 2020.

Antimicrobials	Type of Wards					
	Medicine *n* = 676 *n* (%)	Pediatrics *n* = 389 *n* (%)	ICU *n* = 2 *n* (%)	CCU *n* = 54 *n* (%)	COVID-19 Isolation *n* = 67 *n* (%)	Total *n* = 1188 *n* (%)
**Antibiotics**						
Cephalosporin	365 (54.0)	334 (85.9)	0	18 (33.3)	10 (14.9)	727 (61.2)
First-generation	0	0	0	0	0	0
Second-generation	17 (2.5)	35 (9.0)	0	2 (3.7)	0	54 (4.6)
Third-generation	349 (51.6)	299 (76.9)	0	16 (29.6)	10 (14.9)	674 (56.7)
Fourth-generation	0	1 (0.3)	0	0	0	1 (0.1)
Macrolide	238 (35.2)	7 (1.8)	0	11 (20.4)	9 (13.4)	265 (22.3)
Penicilin	101 (14.9)	53 (13.6)	2 (100)	19 (35.2)	3 (4.5)	178 (15.0)
Aminoglycoside	4 (0.6)	103 (26.5)	1 (50.0)	0	1 (1.5)	109 (9.2)
Quinolones	50 (7.4)	2 (0.5)	0	0	0	52 (4.4)
Tetracycline	47 (6.9)	0	0	2 (3.7)	7 (10.4)	56 (4.7)
Carbapenems	26 (3.8)	14 (3.6)	1 (50.0)	1 (1.8)	2 (3.0)	44 (3.7)
Oxazolidinone	2 (0.3)	10 (2.6)	0	0	0	12 (1.0)
Glycopeptides	0	10 (2.6)	0	0	0	10 (0.8)
Nitroimidazoles	11 (1.6)	1 (0.3)	0	0	0	12 (1.0)
**WHO AWaRe classification antibiotics**						
Access	145 (21.4)	153 (39.3)	2 (100.0)	19 (35.2)	10 (14.9)	329 (27.7)
Watch	547 (80.92)	355 (91.3)	1 (50.0)	25 (46.3)	21 (31.3)	949 (79.9)
Reserve	2 (0.3)	10 (2.6)	0	0	0	12 (1.0)
**Antiviral**						
Acyclovir	3 (0.4)	0	0	0	0	3 (0.2)
Adefovir	1 (0.1)	0	0	0	0	1 (0.1)
Zidovudine	1 (0.1)	0	0	0	0	1 (0.1)
Valacyclovir	1 (0.1)	0	0	2 (3.7)	0	3 (0.2)
Favipiravir	6 (0.9)	0	0	0	2 (3.0)	8 (0.7)
**Antiparasitic drug**						
Ivermectin	28 (4.1)	0	0	2 (3.7)	0	30 (2.5)

**Table A2:** Algorithm for disease severity category in suspected COVID-19 patients.

Classification	Clinical Syndromes	Definition		Absent
Mild	COVID-19-like symptoms	Fever, cough, sore throat, fatigue, headache, muscle pain, anorexia, diarrhea, nausea or vomiting		Pneumonia or difficulty breathing
Moderate	Pneumonia	**Adolescent or adult:** (Any 1) 1. If x-ray conducted: Suggestive of pneumonia 2. If x-ray not conducted: Clinical sign of pneumonia: Fever (≥100.4 °F or ≥38 °C) + any respiratory symptom (cough or difficulty breathing) + decreased breath sound or rales 3. Physician diagnosed pneumonia	**Child** (≤5 years): (Any 1) 1. If x-ray conducted: Suggestive of pneumonia 2. If x-ray not conducted: Clinical sign of pneumonia: Cough or difficulty breathing + fast breath (<2 m: ≥ 60; 2–11 m: ≥ 50; 1–5 y: ≥40) +/or chest indrawing 3. Physician diagnosed pneumonia	**Adolescent or adult:** Respiratory rate: <30 Oxygen = 0 **Child:** Severe pneumonia
Severe	Severe pneumonia	**Adolescent or adult:** (Any 1) 1. Pneumonia + fever + any one: Resp. rate (≥30 breath/min) or oxygen support 2. Physician diagnosed severe pneumonia	**Child** (≤5 years): (Any 1) 1. Pneumonia + cough/difficulty breathing + any one danger sign: central cyanosis or oxygen support or stridor or inability to drink/lethergy/unconsciousness/convulsions 2. Physician diagnosed severe pneumonia	
Critical	Sepsis	**Adult:** (All 3) 1. Suspected or proven infection: Abnormal temp: Fever (low/high) + Neutrophil (high: >75) or ESR (high: >20) or WBC: (high: >12 k or low: < 4 k) or lymphocyte (low: <20) 2. Fast HR (pulse rate- ≥ 12 y: >100) 3. Altered mental status (2–5) or Oxygen support or Thrombocytopenia (Platelet: <50,000/cmm)or Physician diagnosed sepsis	**Child:** (All 3) 1. Suspected or proven infection: Abnormal temp: Fever (low/high) + Neutrophil (high: >75) or ESR (high: >20) or WBC: (high: >12 k or low: <4 k) or lymphocyte (low: <20) 2. HR (pulse rate- 0–29 d: >180, 30 d-1 y: >160, 1–5 y: >140, 5–12 y: >90) 3. Altered mental status (3–5) or Oxygen support or Thrombocytopenia (Platelet: <50,000/cmm)or Physician diagnosed sepsis	
	ARDS	Physician diagnosed ARDS		
	Septic shock	Physician diagnosed septic shock		
	Required ICU or mechanical ventilation	ICU or Mechanical ventilation required		
